# Enhancing the cost-effectiveness of mental health services in Georgia-research proposal

**DOI:** 10.1192/j.eurpsy.2025.1658

**Published:** 2025-08-26

**Authors:** N. Zavradashvili, G. Matiashvili

**Affiliations:** 1 School of Health Sciences, The University of Georgia, Tbilisi, Georgia

## Abstract

**Introduction:**

In Georgia, the psychiatry system is predominantly institutionalized, with limited community-based services and inadequate funding for psycho-social rehabilitation. Government finds it challenging to refocus psychiatry care towards deinstitutionalization, often failing to understand the high importance of such a step and the reasoning behind turning it. This research proposal aims to explore strategies to enhance the cost-effectiveness of mental health services in this context.

**Objectives:**

Assess the current landscape of psychiatry services in a middle-income country, focusing on institutional care versus community-based services. Identify barriers and challenges faced by key actors in transitioning towards community-oriented psychiatry care. Investigate the cost-effectiveness of community mental health interventions compared to institutional care, considering long-term outcomes and societal impacts. Develop evidence-based recommendations to advocate for policy changes and resource allocation towards community-oriented services.

**Methods:**

Literature Review: Conduct a comprehensive review of existing literature on cost-effective analyses and community interventions in middle-income countries.Policy Analysis: Examine existing mental health policies and budget allocations to identify gaps and opportunities for reallocating resources toward community-based care. Stakeholder interviews: interview policymakers, mental health professionals, and patients to collect information from different perspectives. Quantitative Analysis: Use health economics methods to analyze the cost-effectiveness of community-based mental health services compared to institutional care.

**Results:**

Insights into the economic impact of transitioning towards community-oriented mental health services in middle-income countries. Policy recommendations aimed at increasing investment in community-based interventions and resocialization programs. Increased awareness among key actors about the long-term benefits and cost savings associated with community-oriented mental health care. Improved understanding of societal attitudes and barriers towards mental health reform.

**Conclusions:**

Key discussion points include: How can advocacy efforts be reinforced to persuade governments that community mental health care is more affordable and beneficial? What obstacles might stand in the way of expanding community-based services, and how might they be overcome? How can stakeholders work together to guarantee resocialization programs receive ongoing financing and support? What role can international partnerships and collaborations play in supporting mental health reform in Georgia? By addressing these critical questions, this research proposal seeks to provide a roadmap for enhancing the cost-effectiveness and accessibility of mental health services in Georgia and similar middle-income contexts.

**Disclosure of Interest:**

None Declared

